# Methodology to create 3D models of COVID-19 pathologies for virtual clinical trials

**DOI:** 10.1117/1.JMI.8.S1.013501

**Published:** 2021-01-04

**Authors:** Sunay Rodríguez Pérez, Johan Coolen, Nicholas W. Marshall, Lesley Cockmartin, Charlotte Biebaû, Jeroen Desmet, Walter De Wever, Lara Struelens, Hilde Bosmans

**Affiliations:** a KU Leuven, Medical Physics and Quality Assessment, Leuven, Belgium; b SCK CEN, Radiation Protection Dosimetry and Calibration, Mol, Belgium; c UZ Gasthuisberg, Department of Radiology, Leuven, Belgium

**Keywords:** COVID-19 pathologies, voxel phantoms, mesh modeling, COVID-19 imaging, computed tomography segmentation, computer simulations

## Abstract

**Purpose:** We describe the creation of computational models of lung pathologies indicative of COVID-19 disease. The models are intended for use in virtual clinical trials (VCT) for task-specific optimization of chest x-ray (CXR) imaging.

**Approach:** Images of COVID-19 patients confirmed by computed tomography were used to segment areas of increased attenuation in the lungs, all compatible with ground glass opacities and consolidations. Using a modeling methodology, the segmented pathologies were converted to polygonal meshes and adapted to fit the lungs of a previously developed polygonal mesh thorax phantom. The models were then voxelized with a resolution of $0.5\times 0.5\times 0.5\;{\mathrm{mm}}^{3}$ and used as input in a simulation framework to generate radiographic images. Primary projections were generated via ray tracing while the Monte Carlo transport code was used for the scattered radiation. Realistic sharpness and noise characteristics were also simulated, followed by clinical image processing. Example images generated at 120 kVp were used for the validation of the models in a reader study. Additionally, images were uploaded to an Artificial Intelligence (AI) software for the detection of COVID-19.

**Results:** Nine models of COVID-19 associated pathologies were created, covering a range of disease severity. The realism of the models was confirmed by experienced radiologists and by dedicated AI software.

**Conclusions:** A methodology has been developed for the rapid generation of realistic 3D models of a large range of COVID-19 pathologies. The modeling framework can be used as the basis for VCTs for testing detection and triaging of COVID-19 suspected cases.

## 1 Introduction

During the ongoing COVID-19 outbreak, thoracic imaging by means of computed tomography (CT) and/or planar chest x-ray (CXR) is being used as a key tool in early diagnosis and disease monitoring, in particular, to establish severity and clinical progress. Reverse transcription polymerase chain reaction (RT-PCR) results are considered the gold standard for diagnosis of COVID-19, but as thoracic imaging has been able to positively confirm cases after false-negative RT-PCR tests,^1^ this is often used in clinical practice. Access to chest CT or CXR is especially useful where there is a high influx of symptomatic patients, a shortage of tests, and potentially long waiting times for the results.

Although CT is the preferred imaging modality for diagnosing COVID-19 patients, CXR is widely used for follow-up of the disease, with CXR performed daily on patients in the intensive care unit. CXR systems have many logistical advantages over CT, among them their wide availability and short examination times. A CT examination requires transport of infectious patients to dedicated rooms followed by extensive and time-consuming decontamination of the system. In contrast, portable CXR devices can be transported to areas designated for COVID-19 patients within the hospital and even to external locations including care homes. Early research has shown that the sensitivity of CXR for COVID-19 is in the range of 70%,^2^ whereas a figure of ∼90% has been found for CT.^3^

The rationale for this study is that an attempt should be made to improve the performance of CXR when used in the diagnosis of COVID-19. There are different technical approaches available for this, including new detectors, improved antiscatter rejection techniques, rib suppression software, and/or dual-energy acquisitions. Given the urgency of the situation, we have developed a virtual clinical trial (VCT)^4^^–^^6^ platform that will allow for dedicated optimization studies without the difficulties associated with clinical studies on (critically ill) patients. The objective of this work was, therefore, to create a series of COVID-19 models that can be used to generate simulated CXR images for future VCTs. The method for producing the COVID-19 lesion models is described in detail; nine models were then evaluated for realism by radiologists. Alongside the radiologist rating, an Artificial Intelligence (AI)-based software tool, developed for real patient cases, was used to assess the virtual cases for the presence of COVID-19 pathology. This tool was used as an extra realism estimate in addition to the realism scores of radiologists.

## 2 Materials and Methods

### 2.1 Realistic Anthropomorphic Flexible Phantom

Modeling started from the realistic anthropomorphic flexible (RAF) phantom, a full body male phantom developed by Lombardo et al.^7^ using polygonal mesh modeling. This type of geometrical representation is widely used in computer graphic modeling^8^ and describes the phantom with a collection of polygons that share vertices and edges fulfilling certain rules.

**Fig. 1:**
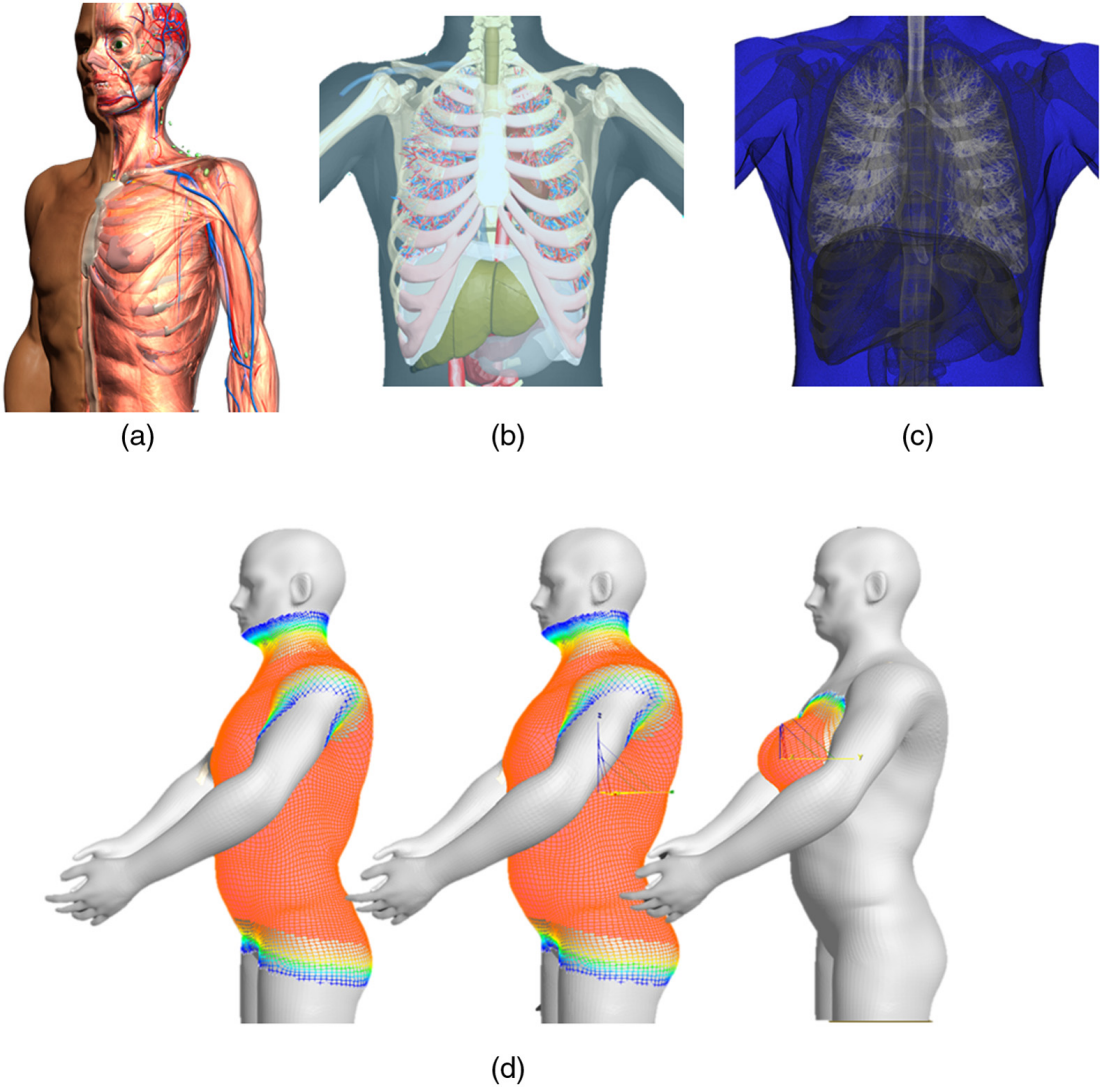
(a) Picture of the RAF mesh phantom; (b) mesh version of the RAF phantom adapted for chest imaging applications; (c) voxelized version of the RAF phantom; and (d) different versions of the RAF phantom representing (from left to right) the standard male, the modified overweight male, and a female.

The RAF phantom had been validated against the ICRP Publication 110 phantom^9^ and provides a detailed depiction of human anatomy [Fig. 1(a)]. The polygonal mesh format of the phantom allows for further modification of anatomy and posture. This flexibility is important for the accurate modeling of patient position during radiographic examinations, as this can range from an erect chest posterior anterior (PA) to a bedside anterior posterior examination. For current chest imaging applications, only the organs in the thorax region of the RAF phantom have been developed further. Additional modifications included the modeling of a more detailed lung background^10^ with bronchial trees, pulmonary arteries, and pulmonary veins [Figs. 1(b) and 1(c)].

The RAF phantom represents a human male with a body mass index (BMI) of $24\;\mathrm{kg}/m^{2}$. Additional versions of the phantom were created to represent an overweight ($\mathrm{BMI}=29\;\mathrm{kg}/m^{2}$) and obese ($\mathrm{BMI}=40\;\mathrm{kg}/m^{2}$) male as well as a female version with a BMI of $29\;\mathrm{kg}/m^{2}$ [Fig. 1(d)]. The different body types were created by modifying the external shape of the phantom. Graphic modeling software 3ds Max (Autodesk, USA) was used for this purpose. The soft selection tool was employed as this allows the vertices of the meshes to be deformed in an organic way. Figure 1(d) shows an example of the soft selection; the red color represents the meshes explicitly selected, whereas the remaining colors represent the vicinity meshes. As the selected meshes are transformed (i.e., translated, rotated, and/or scaled), the elements in the vicinity are drawn along smoothly, and this effect decreases with distance or the “strength” of the selection. The internal organs of the phantom were kept unchanged. A study by Lemanowicz et al.^11^ found the level of patient obesity to have the highest correlation with the chest soft tissue thickness; thus, it was considered correct to keep the internal organs unchanged for the first set of models.

### 2.2 Modeling of the Pathologies from CT Scans

Ethical approval was requested for a retrospective study making use of patient CT images; the ethical committee waved any dedicated patient consent. A 3D modeling methodology was implemented to create the COVID-19 disease within the RAF phantom. CT images of patients suspected of COVID-19 confirmed by CT scans were used as reference for the development of the pathology models. The CT scans had been acquired with a low-dose thorax protocol using 120 kVp, 1.2 mm pitch, and 46 mAs (tube current modulation off). CT image voxel size ranged from 0.68 to 1.00 mm and slice thickness was 3 mm.

**Fig. 2:**
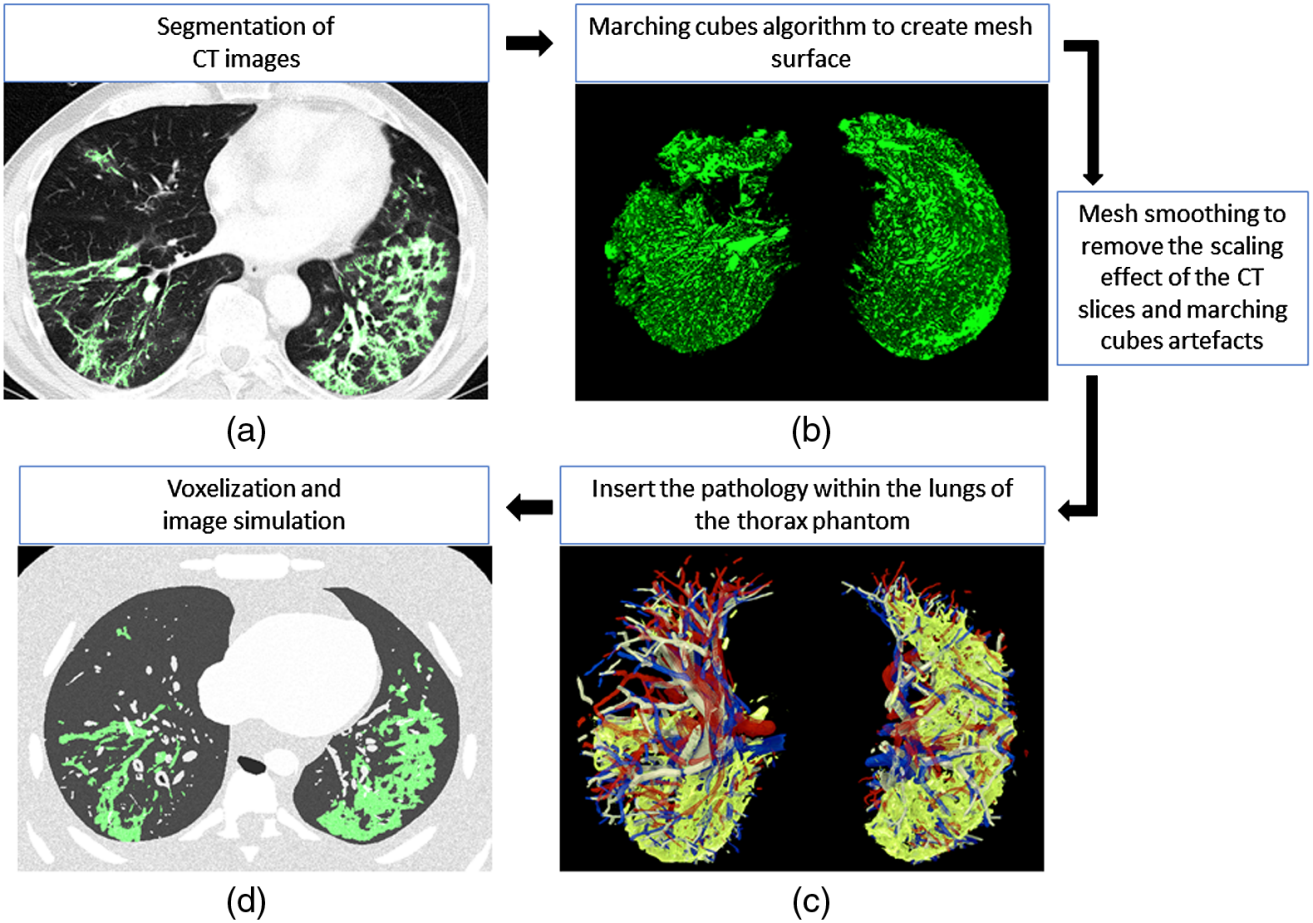
Workflow of the methodology followed to model the pathologies: (a) patient CT slice with segmented pathology (green); (b) 3D surface of the pathology converted to mesh format; (c) slice of the 3D mesh model of the pathology (green) inside RAF phantom lungs; and (d) slice of voxelized RAF phantom with highlighted pathology (green).

A range of cases was selected by a radiologist to ensure that the different stages of the disease would be covered. Areas of ground glass opacities (GGO) and consolidation were segmented manually using ImageJ^12^ [Fig. 2(a)]. The segmentation was carried out by a medical physicist but was closely guided by a radiologist. The segmented pathology was then converted to a binary stack image in which the pathology was colored white and the background black. Next, the marching cubes algorithm^13^ was applied to extract polygonal meshes from the isosurfaces of the three-dimensional pathological structures [Fig. 2(b)]. This conversion from CT voxels to meshes was done using the 3D visualization library in ImageJ.^14^ A resampling factor of 1 was chosen for the marching cubes, so the meshes would replicate the voxel structures without a loss of resolution. The mesh volume of the pathology was then exported as an OBJ file containing the coordinates of the vertices in 3D space; this in turn defined the shape and size of the surface of the segmented pathology.

The OBJ lesion model was imported into 3ds Max, where two steps were used to correct the meshes and generate consistent models. First, the voxel scaling effect due to the segmentation process in anisotropic CT data was smoothed. Artifacts generated by the marching cubes algorithm were removed using the TurboSmooth and Relax modifiers. The latter were sparingly applied to avoid loss in the model volume. Second, the shape of the pathology was modified to fit the RAF phantom lungs, while respecting the volume of the segmented disease [Fig. 2(c)]. This step was carried out using the free form deformation modifier. The deformations applied in this step required manual intervention since the shape of the lung and its pathology changes from case to case. Non-isotropic scaling was utilized. The original spatial distribution of the pathology over the lung was preserved, and changes in the volume ratio disease/lung ($V_{\text{segmented}\_\text{pathology}}/V_{\text{patient}\_\text{lung}}$) were kept below 15%. The set of models represented the typical distributions of the disease: predominantly in the lower lobe, multifocal, peripheral, and bilateral.^15^ To quantitatively assess differences between the segmented lesion and the final model used in the phantom, the Hausdorff distance (HD) was calculated for the mesh models. Meshlab software,^16^ which samples a set of points over the mesh and finds the closest point in the reference mesh, was used.

The modeling methodology enables the representation of different grades of severity of lung involvement by changing the x-ray attenuation in the simulated pathology or by changing its size and distribution. As a proof of concept for the development of different lesion severities from the reference set, the mesh of one of the segmented lesions was modified by changing its size and shape.

An example of a model in which the x-ray attenuation of the pathology was changed to study the impact on lesion detection was also simulated. Finally, the influence of BMI on lesion detection is also illustrated for one disease model simulated in the different BMI and gender realizations of the phantom. These examples are shown in the results (Sec. 3.5).

### 2.3 Creation of Voxelized Phantoms

**Fig. 3:**
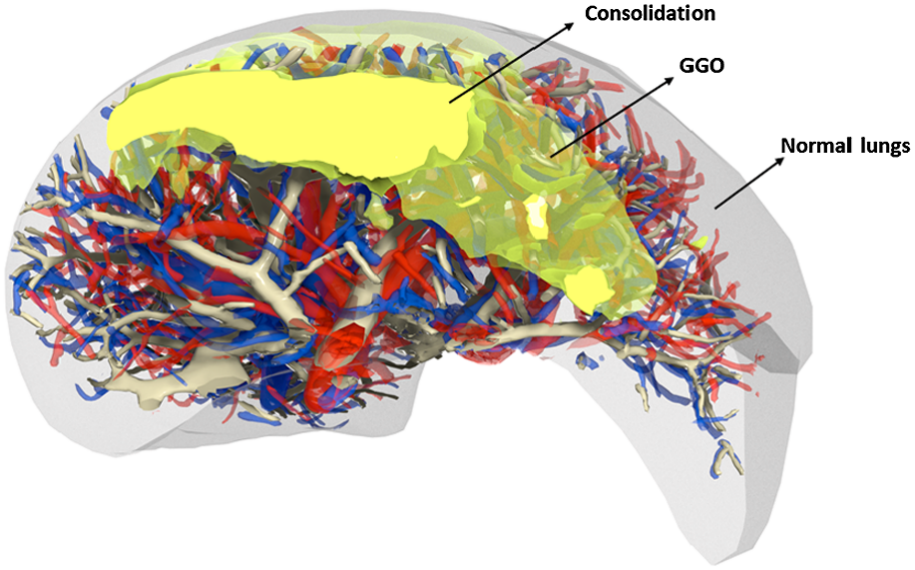
Slice of mesh model of the lung containing GGO and consolidation regions. GGO are regions of increased opacity in the lungs where pulmonary structures are still visible, whereas in the consolidation the pulmonary structures are obscured.

The polygonal mesh models were then exported separately and loaded into voxelization software.^7^ The latter software tool is based on the work of Laine^17^ and is optimized such that the mass and thickness of the organs is preserved. The algorithm uses a conservative eight-separating voxelization method by calculating intersections between the triangles and quads from the meshes with a cube used as the test object.^17^ The voxel resolution used for the models was $0.5\times 0.5\times 0.5\;{\mathrm{mm}}^{3}$, which was a compromise that maintained fine detail within the phantom anatomy and yet kept the computational load reasonably low. Once all of the organs and pathologies were voxelized; they were combined and assigned an ID number using a script developed for ImageJ.^12^ The process started from the thoracic wall and went in an increasing hierarchy to the bones, diaphragm, lungs, heart, up to the pulmonary veins, vessels, and the bronchi (highest priority) [see Fig. 2(d)]: IDs were overwritten where there was superposition. The GGO ID was added after the lungs since GGO represent an area of increased opacity in which bronchial structures and vessels are still visible. The consolidation ID was added last, as consolidations obscure all pulmonary structures within some region^18^ (see Fig. 3).

Finally, the volume ratio of the segmented pathology to that of the patient lung ($V_{\text{segmented}\_\text{pathology}}/V_{\text{patient}\_\text{lung}}$) was compared with the volume ratio of the voxelized pathology model to that of the phantom lung ($V_{\text{final}\_\text{pathology}}/V_{\text{phantom}\_\text{lung}}$). This served as a validation of the method to ensure that the volume ratio of pathology/lung was kept similar, regardless of differences between the patients and the RAF chest phantom.

### 2.4 Generating Radiographic Images Using a Simulation Framework

An imaging chain simulation platform developed in-house was used to generate radiographic images of the models. The simulation platform includes all elements from the imaging chain: x-ray source, patient model, antiscatter grid,^19^ and detector. The simulations combined a ray tracing algorithm^20^ and Monte Carlo simulations using the PENELOPE/penEasy^21^^,^^22^ transport code. The ray tracing was used to create noise free, high-resolution primary projections (i.e., images generated by unscattered photons) in relatively short computation times. PenEasy was used to create scatter images by tracking photons that underwent energy loss or angular deflection before arriving in the detector. The random nature of the Monte Carlo simulations required long computational times to obtain low uncertainty values. To reduce the computational time, pixels of $5\times 5\;{\mathrm{mm}}^{2}$ were used in the scatter image. This was considered sufficient to represent the variations of the scatter radiation since these images do not contain fine details.^23^ The uncertainty of the Monte Carlo calculations was kept below 3%. The materials and proportions of the mixtures used for the different phantom organs were taken from ICRP Publications 89^24^ and 110.^9^ GGO and consolidations are areas of increased levels of x-ray attenuation due to the presence of fluid in the lungs. To obtain realistic pathology densities, the Hounsfield unit (HU) histograms of the corresponding segmented lesions in the CT images were measured and subsequently converted to density ($g/{\mathrm{cm}}^{3}$) using the data of Schneider.^25^ A random distribution of densities was used in the pathology region of the phantom. A random value generator was used to assign different attenuation coefficients to each voxel in the pathology region: each density value was given the probability of the corresponding HU in the CT images. To achieve this, the probability distribution was taken from the normalized HU histogram of the segmented lesion.

Realistic levels of sharpness and noise, relevant to a typical x-ray detector for chest projection imaging, were then applied to the simulated phantom images. Sharpness and noise were quantified using the presampling modulation transfer function (MTF) and the normalized noise power spectrum (NNPS), respectively. These metrics were measured from a Carestream flat panel CsI digital detector with a beam quality of 120 kVp and 9 cm PMMA (i.e., lung equivalent thickness^10^) at the tube exit. The presampling MTF was measured using a version of the edge technique described by Samei et al.^26^ A square tantalum edge test object of dimension $50\times 50\;{\mathrm{mm}}^{2}$ and 1-mm-thickness was placed at the center of the image receptor and oriented manually to obtain an angle of ∼3  deg between the edge and the detector matrix. The MTF images were acquired at a detector air kerma (DAK) of 8  μGy. The NNPS was measured from flat field images acquired at different dose levels, with DAK values ranging from 0.7 to 25  μGy.^27^ A region of 1024×1024  pixels was extracted from the image center and a 2D polynomial fitted to and then subtracted from this region to reduce the influence of large area non-uniformities on the final NNPS.^28^ Half-overlapping regions of interests of dimension 128×128  pixels were then extracted from this region, from which the 2D NNPS was calculated.^29^

The sharpness and noise, as characterized by the MTF and NNPS, respectively, were then applied as follows. The 1D presampling MTF was fitted and expanded to a 2D MTF using weighting matrices described in the literature.^30^ The fast Fourier transform (FFT) of the simulated phantom image was then multiplied with the 2D MTF, and an inverse FFT applied to obtain a noise-free image with a realistic level of blurring.^23^ Image noise was then applied to the blurred images via the NPS following the methods of Båth et al.^31^ and Mackenzie et al.^32^ The method consists of forming a noise image using the different components of the NNPS, i.e., electronic, quantum, and fixed pattern noise. Three random Gaussian white noise images with zero mean and unit standard deviation were weighted by these components. These images were individually multiplied with the blurred phantom images and added to form the total noise image. The final simulated x-ray projection was obtained by summing the noise image and the blurred image. Radiographic images of all of the models were generated at 120 kVp, grid in, and 180 cm source to detector distance, settings commonly used in thorax PA examinations. A DAK level of ∼8  μGy, which is higher than in the clinical protocol, was simulated. A PA exposure setting with grid and a higher dose level were selected to obtain high-quality images, in which the pathology was clearly visualized. Finally, clinical image processing corresponding to an adult thorax examination was applied to the images using image processing software MUSICA (Agfa, Belgium).

The imaging chain simulation was validated by comparing the signal difference to noise ratio (SDNR) values from real and simulated images of a test object consisting of a PMMA block (10 and 20 cm thickness) and an aluminum detail (2 mm thickness). Relative differences between SDNR in the real and simulated images were below 10%. This validation was performed over a range of tube voltages and dose levels.

### 2.5 Assessment of Task Realism

To assess the realism of the pathologies in the RAF phantom, the simulated radiographic images were presented to three thorax radiologists, trained during the outbreak to diagnose COVID-19 suspected patients. A reader study in which the observers were asked to score nine different images for each of the COVID-19 models inserted within the standard BMI male RAF was set up. The score was carried out according to three realism criteria: question 1: realism of the lung background, question 2: realism of the lesions in terms of appearance, and question 3: realism of the lesions in terms of position within the lungs. A five-point scale was used for all criteria: (1) not at all realistic: critical elements that affect the realism, (2) poor: obvious elements that may affect the realism, (3) adequate: minor elements that did not affect the realism, (4) good: minimal unrealistic elements, and (5) very realistic: no unrealistic elements. In addition, the readers were asked to write a description of the pathology that was seen. Images were displayed using Viewdex software.^33^ Contrast and brightness levels could be adjusted if desired, and no time limit was imposed.

As an additional validation of the models, the images were uploaded to the AI software Lunit INSIGHT CXR for COVID-19 (Lunit, South Korea).^18^ This software can detect areas of consolidation or GGO in the chest and is intended to support the interpretation of CXR of suspected COVID-19 cases. The software analyses the images and reports an abnormality score given by the likelihood of the presence of the detected lesion, i.e., low (0% to 15%), moderate (16% to 50%), and high (51% to 100%).

## 3 Results

### 3.1 Pathology Models

**Fig. 4:**
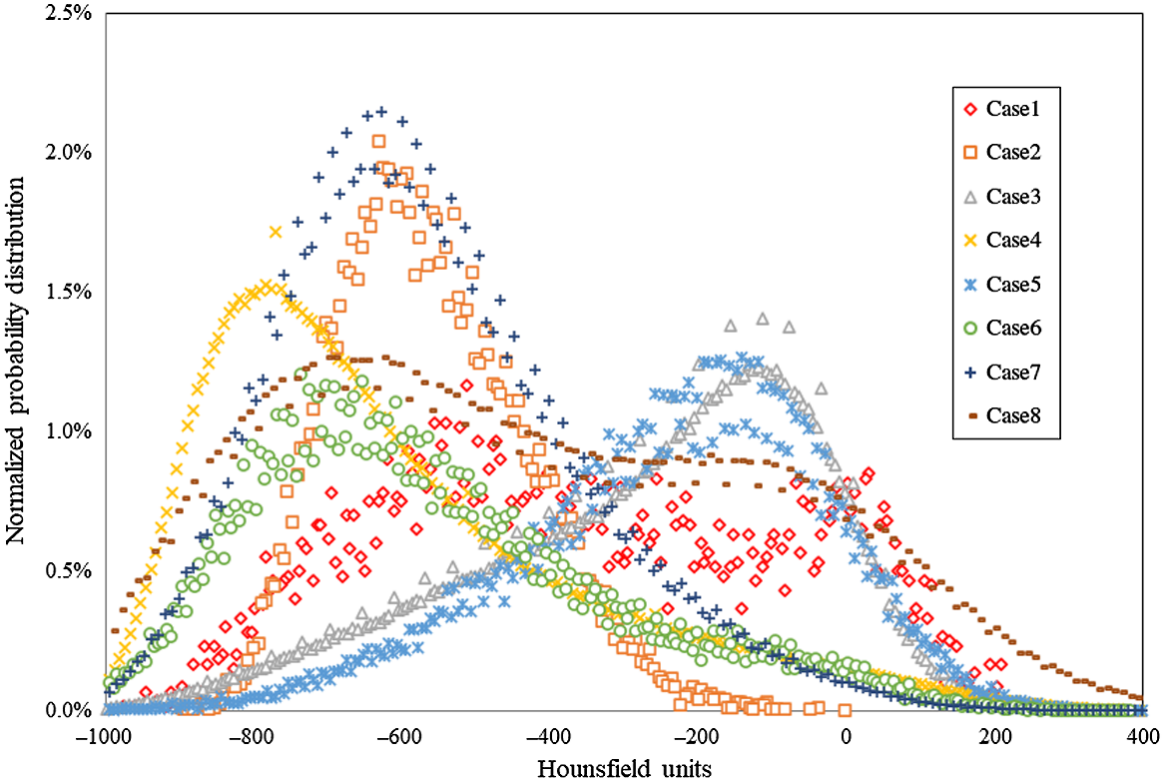
HU distribution within the lesions for the pathologies modeled from the segmentation of CT images (cases 1 to 8).

Nine COVID-19 disease models were created, covering the typical manifestations of lung involvement distribution characteristics of COVID-19 pneumonia. Eight of the models were created from the segmented lesions in CT images and one extra case was created by modifying the mesh of one of the segmented models. The HU histograms obtained for the lesions of the eight segmented CT datasets are shown in Fig. 4. The differences in HU are related to the stage of the disease. Although GGOs lie in the range −800 to −400, higher opacities like consolidations reach 100 HU, consistent with data reported by Lanza et al.^34^

**Fig. 5:**
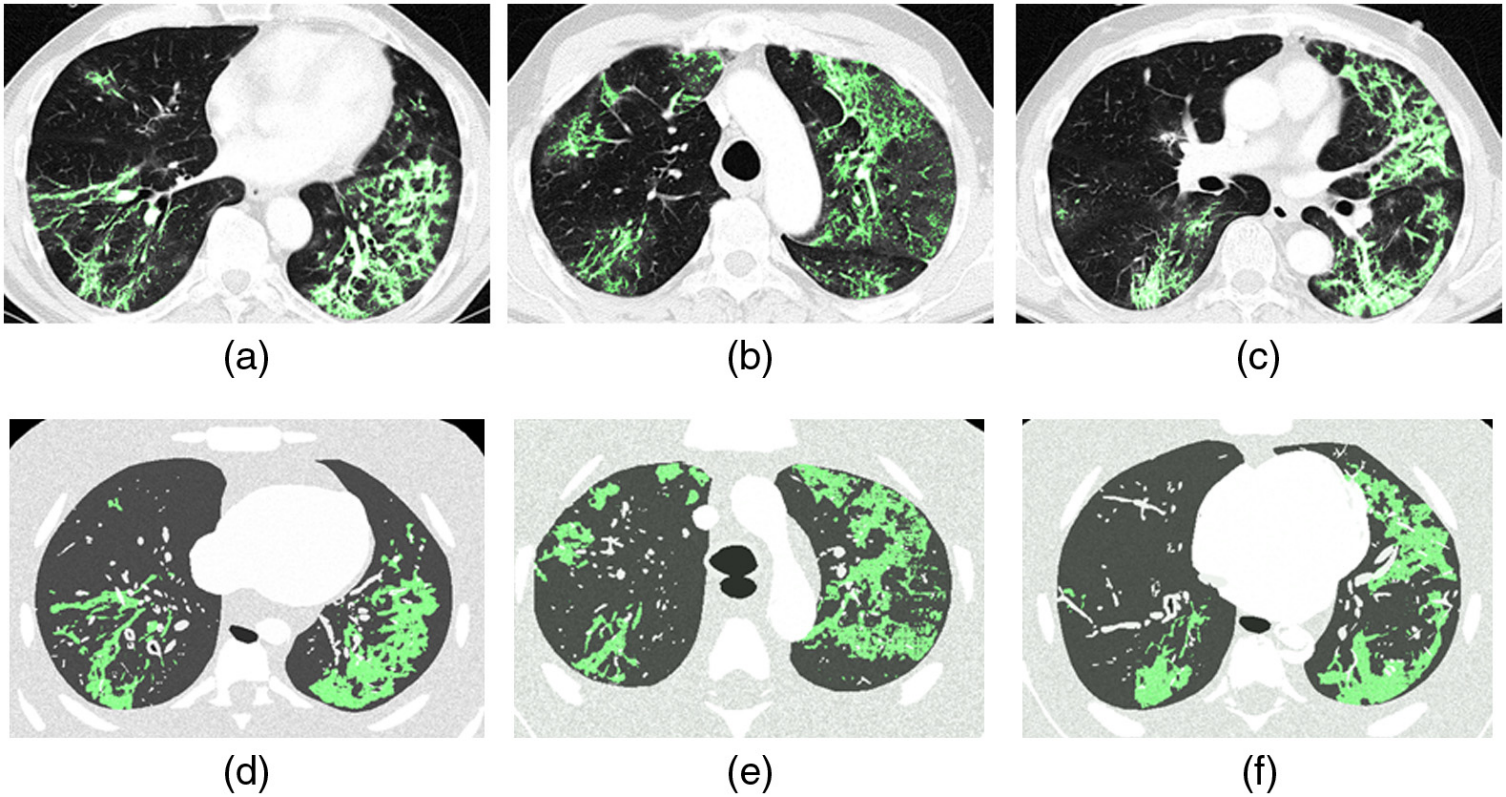
(a)–(c) Comparison of CT slices of a patient (case 4) and (d)–(f) the respective slices of the voxelized RAF phantom. Voxels corresponding to the pathology are highlighted in green.

Figure 5 shows a comparison of different slices of the CT data [(a)–(c)] to the corresponding slice of the voxelized phantom [(d)–(f)] for case 4. The pathology voxels are highlighted in green in all of the images.

**Fig. 6:**
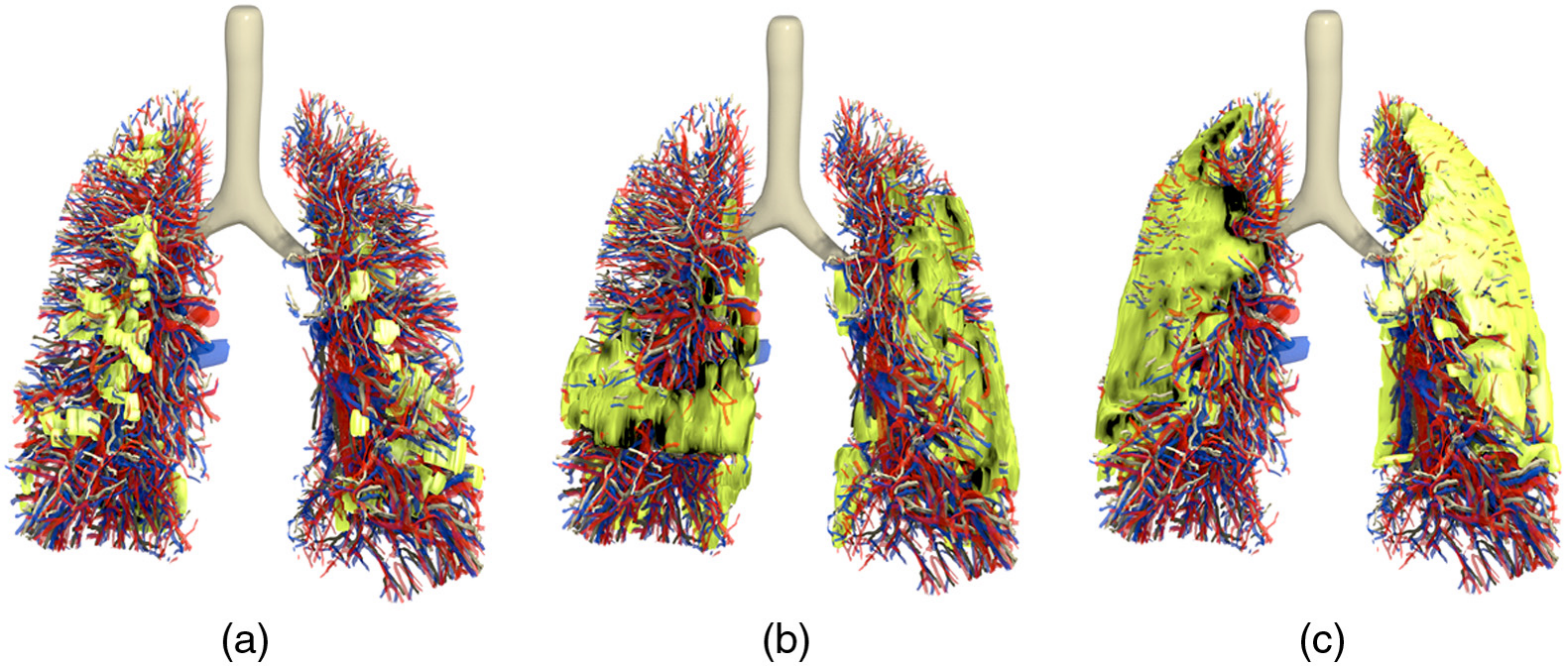
3D mesh models of the pathologies (green) modeled within the lungs of the RAF phantom. Different levels of lung involvement can be seen, from subtle (a) case 6 to more prominent in (b) case 4 and (c) case 7.

Different stages of the disease were modeled, from the more subtle in the initial stage to more advanced. Figures 6(a)–6(c) show the mesh models of the RAF phantom lungs including the pathologies from cases 6, 4, and 7, respectively. As illustrated, the level of lung involvement changes from case to case; the ratios of pathology volume to lung volume of the voxelized versions of these models can be found in Table 1.

**Table 1:** Volume ratios of pathology to lung for the segmented CT images and those for the corresponding phantom models. Relative deviation between the developed models and the patient data is also shown. Mean Hausdorff distance calculation for the mesh models of cases 1 to 8. No CT data are available for case 9 since it is a modified version of case 4.

Case number	$V_{\text{segmented}\_\text{pathology}}/V_{\text{patient}\_\text{lung}}$ (%)	$V_{\text{final}\_\text{pathology}}/V_{\text{phantom}\_\text{lung}}$ (%)	Relative deviation (%)	Mean HD (cm)
1	12.2	13.1	7	0.64±1.02
2	13.2	12.7	−4	0.42±0.53
3	39.0	38.2	−2	0.21±0.31
4	14.0	12.6	−10	0.16±0.34
5	2.6	2.3	−12	0.21±0.29
6	2.6	2.2	−16	0.34±0.66
7	34.6	29.3	−15	0.18±0.24
8	34.2	36.7	7	0.25±0.20
9	—	9.8	—	

### 3.2 Mesh and Volume Comparison

Table 1 shows the volume ratios of pathology/lungs for the developed models. As can be seen, the percentage of lung involvement ranges from 2.2% to 38.2%. The pathology/volume ratios for the segmented CT images and the relative differences compared with the voxelized models are also shown. As observed, maximum differences between the initially segmented lesion and the corresponding final phantom version stayed below 16%. Volume ratios were usually smaller when the pathology was placed in the phantom. No comparison is shown for case 9 since this model is a modification of case 4; thus, no reference volume ratio is available.

The mean HD between the original mesh and the mesh adapted to the RAF phantom can be found in Table 1. Mean HD values were below 0.64 cm.

### 3.3 Simulated Radiographic Images

**Fig. 7:**
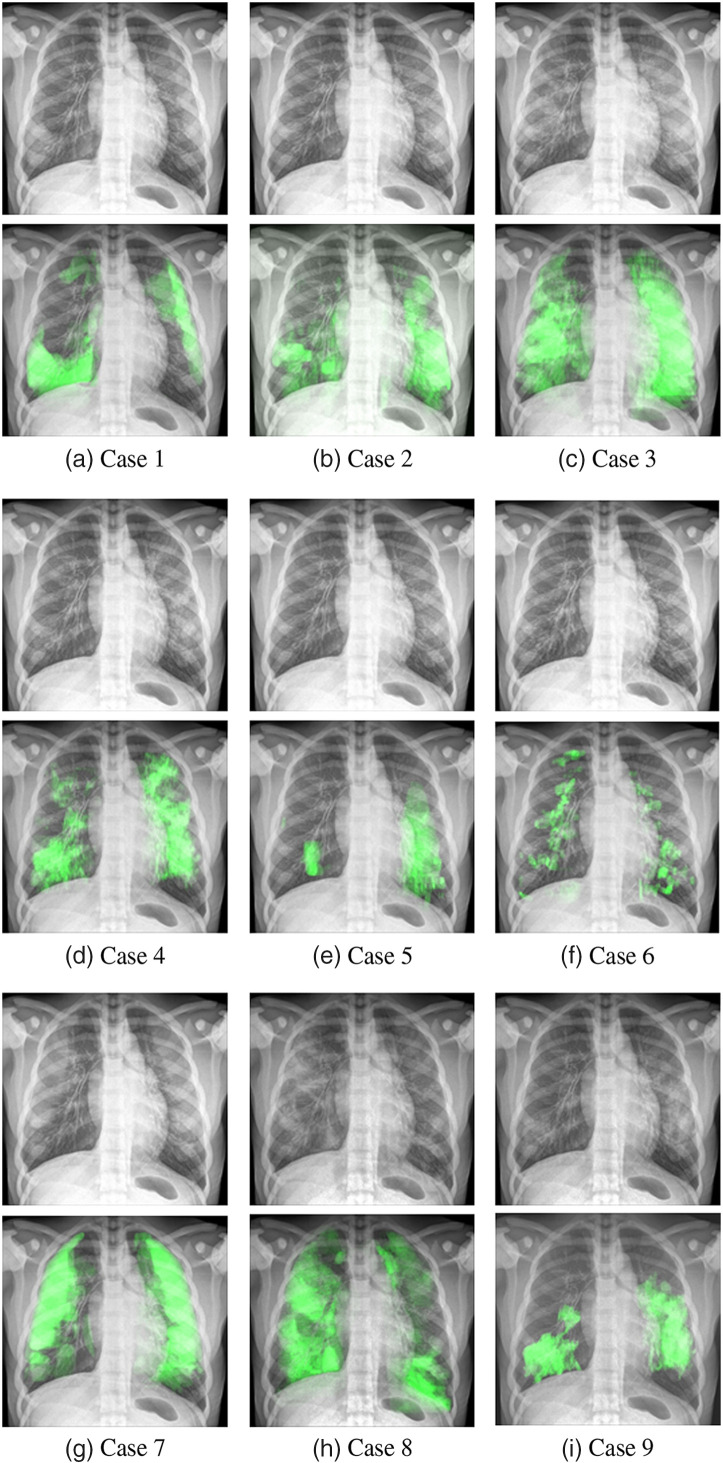
Simulated images of the RAF phantom including the COVID-19 models created. From (a)–(i) cases 1 to 9, respectively. Below each image, an equivalent version with pathology regions highlighted in green is shown.

Figures 7(a)–7(i) show the set of simulated radiographic images of the RAF phantom featuring the nine pathology models. For each case, a projection is also shown with the voxels containing the pathology highlighted in green. As observed, the pathologies in all cases are bilateral, often located in the periphery of the lungs and involving several lobes. The degree of spread of the disease is easily noticeable in each of the cases.

### 3.4 Assessment of Task Realism

Table 2 presents the percentage of cases marked as at least adequate or at least good for the individual observer and the mean realism criteria of all three observers used in the reader study. For questions 1, 2, and 3 (Q1, Q2, and Q3, respectively), the average percentages of cases marked at least adequate were 100%, 92%, and 96%, respectively. The average percentage of cases marked at least good were 59%, 54%, and 65% for Q1, Q2, and Q3, respectively. The mean value for the three quality criteria was at least adequate for 96% of the cases and at least good for 50% of the cases.

**Table 2:** Percentage of cases rated by radiologists as at least adequate and at least good for each of the realism criteria in the images. Q1: realism of lung background, Q2: realism of lesion (appearance), and Q3: realism of lesion (position).

	Percentage of cases marked at least adequate				Percentage of cases marked at least good			
	Q1 (%)	Q2 (%)	Q3 (%)	Mean Q1–Q3 (%)	Q1 (%)	Q2 (%)	Q3 (%)	Mean Q1–Q3 (%)
Radiologist 1	100	88	88	88	88	50	63	63
Radiologist 2	100	89	100	100	0	67	89	56
Radiologist 3	100	100	100	100	89	44	44	33
Average	100	92	96	96	59	54	65	50

For readers 1, 2, and 3, 92%, 96%, and 100% of all scores given to the images, respectively, were at least adequate; they detected only minor unrealistic elements that did not affect the general realism of the models. Moreover, 67%, 52%, and 59% of all scores were at least good for readers 1, 2, and 3, respectively. A moderate agreement was found between the readers with an intraclass correlation coefficient (ICC) of 0.5 (95% CI=0.10 to 0.84).

Radiologists 1, 2, and 3 detected 78%, 89%, and 89% of all COVID-19 pathologies, respectively. Case 6 was missed by all three readers, and case 5 was missed by reader 1. Although reader 3 had marked an area of opacities in this lung, he was uncertain about its presence. These two cases, in fact, represent the more subtle simulated pathologies. Further investigation of case 5 revealed, that after three PCR tests, this patient was negative for COVID-19, and it represents a rare case where CT and PCR arrived at different conclusions. This highlights the difficulty in the radiological practice where lesions with spatial distributions typical for COVID-19 and HU distributions like COVID-19 cases can in fact be negative.

**Fig. 8:**
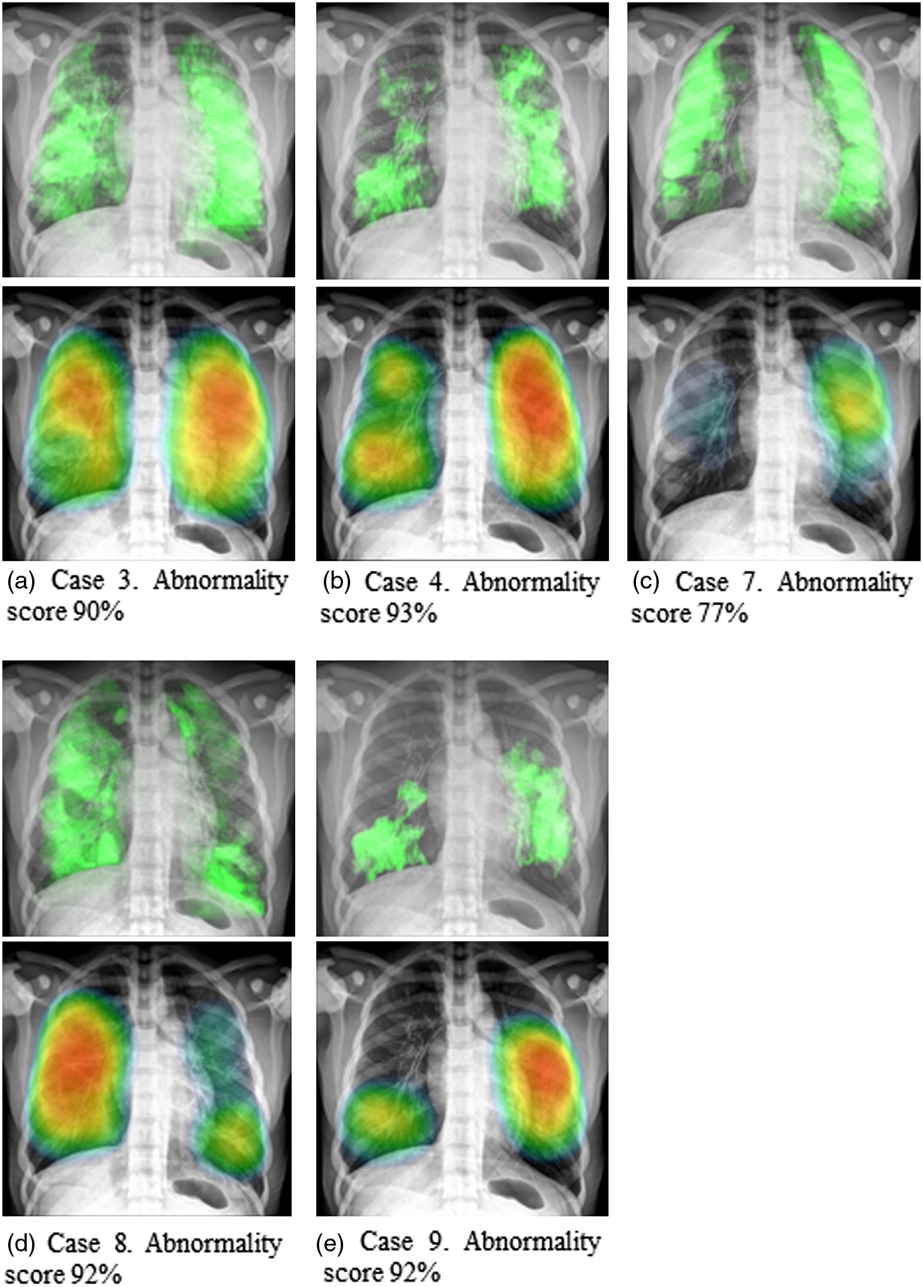
Simulated images for cases (a) 3, (b) 4, (c) 7, (d) 8, and (e) 9. In the upper part is the image with the pathology highlighted in green and below is the corresponding result from the AI software. The heat maps in the bottom images represent the likelihood of the detected lesion to be suggestive for COVID-19.

The AI algorithm was able to identify consolidation areas in 56% of the cases. The algorithm successfully identified pathologic areas in cases 3, 4, 7, 8, and 9. For the rest of the cases, no lesions were detected. Figures 8(a)–8(e) show simulated images with the pathology highlighted in green and the corresponding output from the AI software for cases 3, 4, 7, 8, and 9, respectively. The heat map displayed from the AI software output represents the likelihood of the presence of the lesion as detected by the software. The abnormality scores reported by the AI were 90%, 93%, 77%, 92%, and 92% for cases 3, 4, 7, 8, and 9, respectively.

### 3.5 BMI and Pathology Modifications

**Fig. 9:**
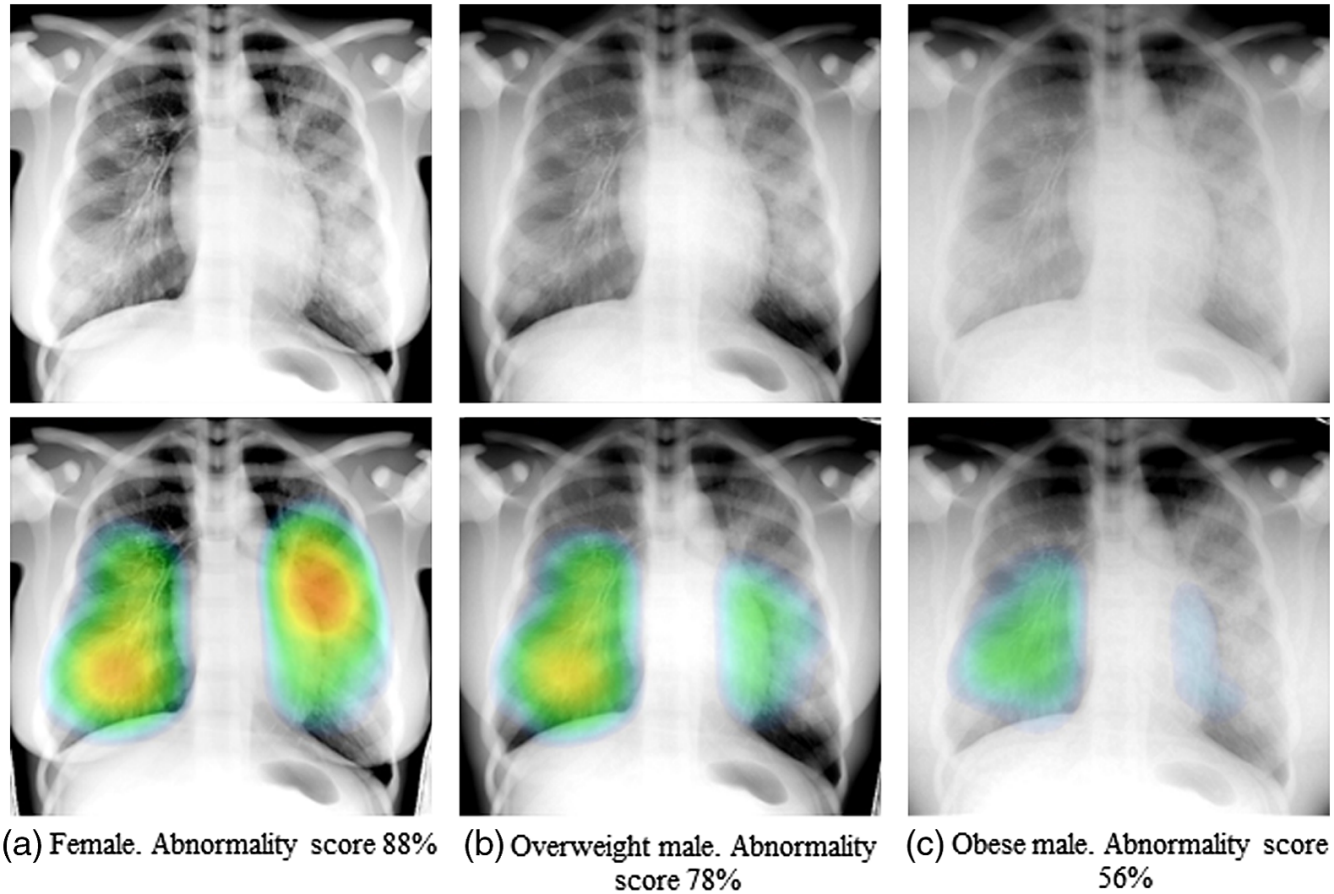
Images of the RAF phantom (with the pathology of case 4) with different body types (a) female (BMI=29); (b) male overweight (BMI=29); and (c) male obese (BMI=40); below each image, the respective output from the AI software is displayed.

Figure 9 presents the images from the RAF phantom with different body types, namely (a) female, (b) overweight male, and (c) obese male. The same pathology, taken from case 4, was used for all of these images [Fig. 7(d)]. As observed, the visualization of the pathology is affected by the increased size of the phantom and by the presence of breasts. These images were analyzed by the AI COVID-19 detection software, and the corresponding output is shown below each case. The different body types had a clear influence on the detection of the pathology, as can be seen in the heat maps of the AI software. The abnormality scores were 88%, 78%, and 56% for the female, male overweight, and male obese, respectively, compared with 93% obtained for the same case in the standard BMI male.

**Fig. 10:**
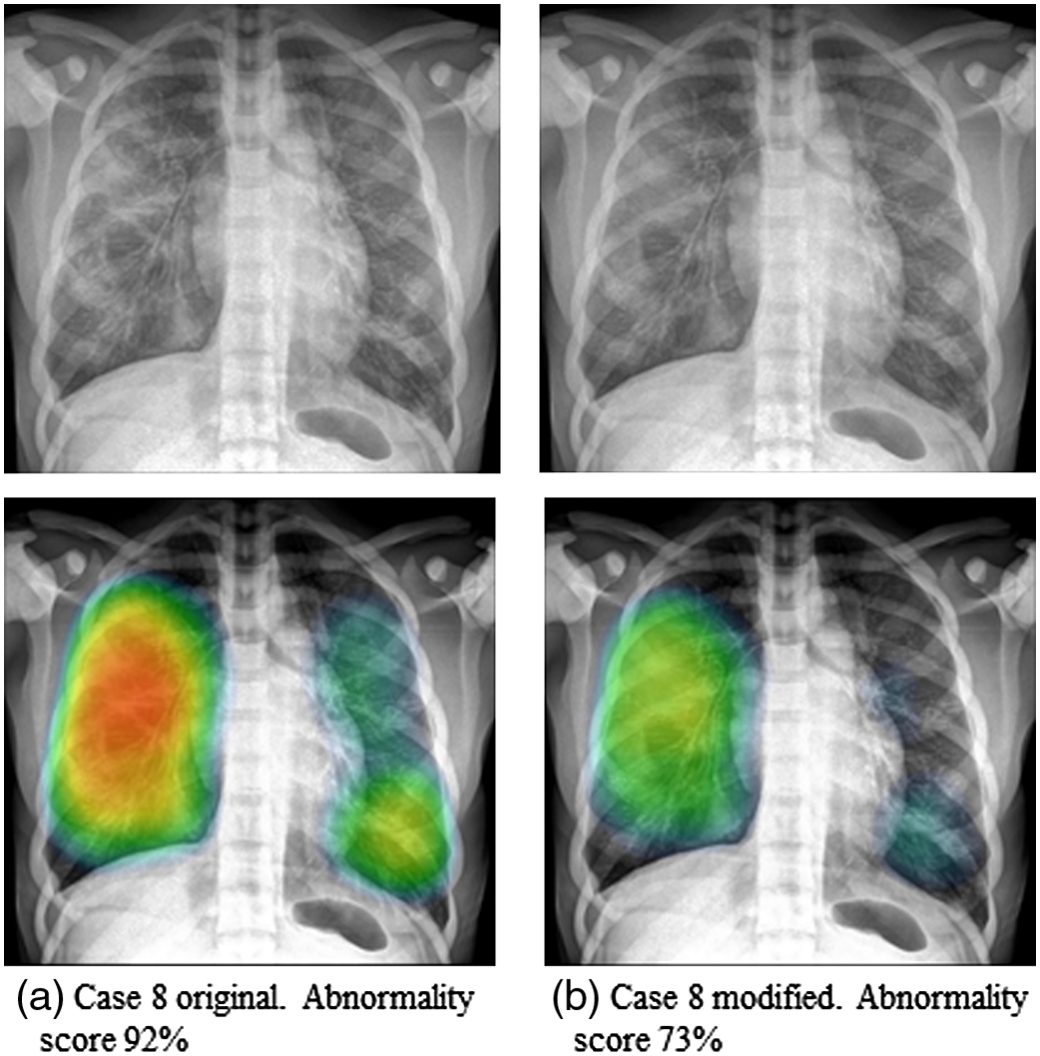
Example of different stages of the disease for case 8. (a) The original models and (b) a more subtle stage with less attenuation in the lungs. The corresponding AI output is shown.

Figure 10 shows the x-ray images of two versions of case 8: (a) represents the original pathology and (b) is a modified version where lower attenuation in the lungs was simulated to represent a more subtle case. These images were uploaded to the AI software for analysis. The respective results are displayed below each simulated image. Heat map intensity falls for the more subtle case, with the abnormality score going from 92% in the original model to 77% in the modified subtler version.

## 4 Discussion

With this work, we have established a methodology for developing computational models of COVID-19 patients. To generate the models presented in this work, it was sufficient to segment the pathology and the lungs of each patient instead of having to segment all of the organs present in the thorax: this reduced the overall modeling time. Depending on the complexity of the pathology and for an experienced developer, segmentation took between 2 to 6 h while 3D modeling ranged from 3 to 6 h. Generation of the input files and images took from 4 to 5 h, which was mostly computational time. The average human input needed to create one model was about 9 h. Having a solid simulation framework and expertise with the creation of computational models played an important role in terms of development time. Tools to automatically segment the pathology from CT images are improving in accuracy and availability, and this could improve the simulation process by making the creation of the models faster. However, this work represents a proof of concept of the creation of COVID-19 patients. If many more models are required for a VCT or any other study, then the use of the latest segmentation tools may have to be explored.

The changes in the ratio of pathology volume to lung volume between the patient and the final model of the phantom remained below 16%. This was considered acceptable because the shape of the pathology was largely preserved; note that 16% represents a deviation of less than one voxel in each direction. The differences can be ascribed to the surface smoothing used to eliminate artifacts from CT, the discretization error introduced by the marching cubes algorithm and the conservative nature of the voxelization algorithm.^35^ The Hausdorff distance, used to compare the meshes of the segmented pathology and the model adapted to the phantom, had mean values below 0.64 cm. These differences were expected since they are attributed to the mesh modification applied when fitting to the lungs of the phantom and the smoothing to eliminate the staircase effect from the CT.

To demonstrate the flexibility of the modeling methodology, the lesion from case 9 was created by modifying the mesh of case 4. This allowed for the creation of an additional model without the need for additional segmentation, but by modifying the shape and size of a pre-existing mesh. When performing this procedure, reported findings from COVID-19 pneumonia and the progression of the disease over time should be verified to ensure correct modeling. Case 9 was included in the validation dataset for the reading study and was classified as at least good by the three readers.

An overweight and obese version of the male phantom and a female version were developed to extend the range of patient types simulated. An example of the effect of BMI and body shape on the pathology from case 4 could be seen when uploading the images to the AI software (see Fig. 9). Another illustration of the potential scope of the method and the models was the realization of different grades of severity of lung involvement. An example (Fig. 10) in which the attenuation in the simulated pathology of case 8 was modified, resulting in a more subtle case, was presented. This modification was reflected in the abnormality score reported by the AI software, which decreased from 92% to 77%. These data suggest that a greater range of BMI values and disease severities can be modeled if required for the VCT.

One of the potential uses of our simulation platform in combination with the COVID-19 models is to assess the influence of x-ray acquisition parameters on the visibility of the models. For example, different tube voltages, x-ray spectral filtering, dose levels, and the presence or absence of antiscatter methods could be investigated by generating images with these characteristics. The models could also be exploited as a means of evaluating new or improved x-ray detector performance by applying the measured characteristics of a new detector, without or with a (new) grid.

The realism of the models was assessed by a reading and scoring experiment in which three thorax radiologists classified the images of the phantoms including the lesions. The mean realism score (mean value of the three scoring criteria) stayed at about 3 (i.e., adequate) for 100% of the cases for reader 2 and 3, whereas for reader 1 this value dropped to 92%. For reader 1, the mean score below 3 was given to case 6, which in fact corresponded to a missed lesion by this reader. On the other hand, 67%, 52%, and 59% of the cases had mean scores above or equal to 4 (i.e., good) for readers 1, 2, and 3, respectively. The fact that the readers missed some of the pathologies is considered acceptable as these cases had very subtle lesions, and it was probably expected that they would be missed on CXR. This is consistent with the modeling of realistic pathologies, but it also shows the need for optimization of planar x-ray imaging for the detection of COVID-19 if high sensitivity is required, for example, if CXR would be used for triage. Although the percentage of cases with mean scores 3 or above (adequate) is similar for the three readers (92% to 100%), the ICC showed only a moderate agreement between the readers. This is due to the differences in the scores of the single cases, given by the readers’ subjectivity and interpretation for this type of study. Additional validation was performed using the Lunit AI algorithm for the detection of COVID-19 disease in CXRs. The algorithm was applied to the same phantom images and was able to identify 56% of the cases.

A major advantage of using models for VCT type studies is that the ground truth of the models is always known: the exact position and attenuation of the pathology is known. The VCT approach can be used for several types of studies. (1) It was shown that at least the current AI algorithm could be tested for sensitivity in our cases. (2) New technologies could also be investigated, for example, dual energy imaging, a technique with good potential for this type of applications since bone structures can be subtracted from the images, providing a clearer view of the lung field. The 3D models can also be used in VCTs carried out using other imaging modalities such as CT and to compare modalities. Case 5, in particular, highlights the potential of VCTs to study sensitivity and specificity of imaging devices. The 3D pathology models developed during this study can be shared upon request with other research groups working in the fight against COVID-19.

Current limitations of the modeling include the lack of anatomical variation in the thorax phantom except for the different versions representing thicker patients. Additionally, the method involves several manual steps, some of them requiring a steep learning curve for less experienced developers. An example is the adaptation of the pathology inside the existing phantom, which can be time-consuming and requires manual intervention. A possibility for speeding up the creation of the models is using automatic segmentation software to identify the pathology in the CT images.^34^^,^^36^

A study using a similar methodology has been recently published by Abadi et al.^37^ In their paper, additional structures within the underlying lung parenchyma were simulated by enlarging the size of the pulmonary lobules to represent crazy paving regions. This can also be implemented in the RAF model if required; however, crazy paving regions are barely visible in plain radiography, which is the current focus for the models created in this work. For the more common manifestations of the disease like GGO and consolidations, Adabi et al. combined fluid with the texture in secondary pulmonary lobules to match the mean linear attenuation coefficient measured for the segmented abnormalities. In this work, we simulated the variation in x-ray attenuation within a diseased region using the HU histogram obtained in the segmented pathology.

## 5 Conclusions

This work has described a method of creating a set of 3D models to represent COVID-19 related lung pathologies. Segmentation and polygonal mesh modeling techniques were used to create nine models from image datasets of CT confirmed COVID-19 patients. The pathologies were then adapted for inclusion within an existing anthropomorphic phantom and simulated radiographic images of the phantom and pathology were generated. The realism of simulated radiographs of the pathologies was assessed by three radiologists and demonstrated by the implementation in an AI software package for COVID-19 detection. These models can form the basis for optimization studies of CXR for COVID-19 imaging using VCTs, but they could also be used in other imaging applications including CT.
